# Ringworm and Its Cure

**Published:** 1906-06-30

**Authors:** J. Stafford Mellish


### THE GENERAL PRACTITIONER'S COLUMN.

[Contributions to this Column are invited, and if accepted will be paid for.]

### RINGWORM AND ITS CURE.

By J. STAFFORD MELLISH, M.R.C.S., L.R.C.P.

HAVING read a good deal in the various medical papers lately about ringworm and its cure, especially by the *x*-rays, this subject became somewhat naturally a topic of conversation when some medical friends of mine joined me at an informal meeting recently. I was more than surprised to find that none of them had used or even heard of a method which I thought was somewhat common; I then remembered that my partner, a young man from a leading London hospital who has recently held all the house appointments, had never seen it used previous to joining me last January, and he is more than pleased with the method and says he has never seen such good results with any other treatment, including that by *x*-rays. It must have been somewhere about '98 that I first used the treatment I am about to describe, and living in a grass country as I do, I may say that I see a lot of ringworm cases—often being called to a family with from four to eight members of it affected in different places; caught usually in the first instance from cattle and transmitted from one member of the family to another. I take it that for practical purposes the perfect desideratum is a remedy that will find access to the hair papilla without killing it, and yet be sufficiently strong to kill all seeds of the trouble. In formic aldehyde (40-per-cent. solution) I think we nearly find what is wanted; at any rate, I am in a position to say that in the last five years I have treated between 250 and 275 cases and only in some 20 cases have I had to give more than one application, and only in seven cases have I had to give three; these latter were all cases with a plentiful supply of hair and where the patients or their parents absolutely refused to have it cut off. When one reads in this week's (June 16) *Lancet* that under Dr. Sabourand the cost of the cure of ringworm by *x*-rays has been reduced from 2,000 francs per case to 260, and when one finds that a 1-pound bottle of "Formalin," costing 1s. 8d., is sufficient to cure some 20 to 50 cases without being very careful of it, one's thoughts turn to unnecessary expense at hospitals. For practical purposes one may divide the cases of ringworm into:—

1. Ringworm of body—Tinea circinata.
2. Ringworm of scalp—Tinea tonsurans.
3. Ringworm of beard—sycosis.
4. Dhobie itch—Tinea marginata.

Of these the first are by far the easier cases to cure, and it will be found that by taking a pad of wool or tow and saturating the end of it with "Formalin" and well rubbing the diseased patch for from 1 to 2 minutes, the greater number of cases will dry up and scale off and disappear with no further trouble. In all cases of ringworm where there are scabs and thick scales I make the patient either use some bread poultices for a night, or some alkaline lotion to remove the scabs before treating with formalin, and my custom after treating is to cover the patch with some ointment; yellow oxide of mercury suits well in children and skin cases in adults, while if the case is one in the hair or beard of an adult I find the ung. hyd. nit. dil., B.P., very satisfactory.

In all cases that have reached a more advanced stage and are attempting to cure themselves by pustules, I have them well scrubbed with soap and water and apply formalin as in the previous cases, afterwards using an ointment and in all cases in heads, or beards, I have the hair cut short, if possible, at any rate round the patch, and proceed as in the former case; by clipping all the hair from the head one is enabled to judge better of the diseased area. Since adopting this treatment I have really had brilliant results, and in no single case have I found it necessary to carry out that painful process of depilation.

Naturally one must carry out the ordinary prophylactic measures of ordering a new paper lining every day in the hats of children, and in many cases having the hat in use burnt at starting. A point well worth bearing in mind, in ringworm about the hands and wrists is to have the wristbands and cuffs of clothes thoroughly disinfected and then cover the seat of disease if on the wrist, with ointment lint and bandage; if on the back of the hand, plenty of ointment smeared on it and an old white kid glove with the fingers cut off can be worn at work. I have noticed that some of the cases treated seem to dry up and die at once, especially those not situated on hairy areas, while others become inflamed and form a kerion; following that form of nature's cure very closely, I am disposed to think that the method of cure in great measure depends on the amount of rubbing, those cases where most rubbing is done becoming more inflamed than those which are let off more lightly. I have tried to make certain of this by rubbing some places on the same patient more than others. However, the practical point is to rub sufficiently to cure.

Some years ago there was a Dr. Dene living near Taunton who had a more than local reputation for the cure of ringworm, and he was wont to say, when he could be induced to talk on the subject, which was seldom, "It's the rubbing that does it;" as far as I was ever able to make out, his treatment was oil of cade and alcohol rubbed in alternately, but he always refused to say, and I believe on one occasion was interrogated on the subject by the Medical Council or some other ruling body, but in his sturdy West-country way, the old gentleman offered to impart his knowledge providing he was pensioned! Needless to say, his secret, whatever it was, did not become public property.

Personally, I think that the success depends to a certain extent on the rubbing, which opens up the mouths of the follicles round the hairs and other pores in the skin, thereby permitting the extremely pungent vapour of formalin to penetrate the follicles; on account of this pungency it is wise to blindfold small children when applying the remedy.

The only objection possible to find with the treatment is the temporary smarting, which is, however, of very short duration (about ten minutes) and in no case have I found the hair follicles killed or the colour of the hair changed.

In those chronic scurfy conditions of the scalp following ringworm I find a good rubbing with formalin disinfects the hair, and yellow oxide ointment well rubbed in to the roots for a week completes the cure.

As regards dhobie itch—a disease mostly met with in the East—I have only seen one case, which was in a London hospital I happened to be visiting and had no opportunity of trying the treatment, but as far as I can see there is no reason why it should not be as successful in this form of the trouble as it is in others.

